# Toxicity and Environmental Risk Assessment of Polycarbamate and Its Main Components to Marine Algae and Crustaceans

**DOI:** 10.3390/ijms24044183

**Published:** 2023-02-20

**Authors:** Toshimitsu Onduka, Kazuhiko Mochida

**Affiliations:** Hatsukaichi Field Station, Fisheries Technology Institute, Japan Fisheries Research and Education Agency, 2-17-5 Maruishi, Hatsukaichi 739-0452, Hiroshima, Japan

**Keywords:** dithiocarbamate fungicide, algal growth inhibition test, crustacean immobilization test, species sensitivity distribution, degradation product, dimethyldithiocarbamate

## Abstract

Polycarbamate is commonly used as an antifoulant coating on fishing nets in Japan. Although its toxicity to freshwater organisms has been reported, its toxicity to marine organisms is currently unknown. We conducted algal growth inhibition and crustacean immobilization tests to assess the effects of polycarbamate on marine organisms. We also evaluated the acute toxicity of the main components of polycarbamate, namely, dimethyldithiocarbamate and ethylenebisdithiocarbamate, to algae, which are the most sensitive tested organisms to polycarbamate. The toxicities of dimethyldithiocarbamate and ethylenebisdithiocarbamate partially explain that of polycarbamate. To assess the primary risk, we derived the predicted no-effect concentration (PNEC) for polycarbamate in a probabilistic manner using species sensitivity distributions. The 72 h no observed effect concentration (NOEC) of polycarbamate to the alga *Skeletonema marinoi–dohrnii* complex was 0.45 μg/L. The toxicity of dimethyldithiocarbamate may have contributed up to 72% of the toxicity observed for polycarbamate. The fifth percentile of hazardous concentration (HC_5_) derived from the acute toxicity values was 0.48 μg/L. Comparison of previously reported environmental polycarbamate concentrations in Hiroshima Bay, Japan, to the PNEC estimated using the minimum NOEC and HC_5_ suggest that polycarbamate currently poses a high ecological risk. Therefore, reducing the risk by restricting polycarbamate use is necessary.

## 1. Introduction

Some antifouling compounds have been used on fishing nets and ship hulls as alternatives to organotin antifoulants, which are restricted compounds [1]. Among these antifouling compounds, polycarbamate (PC) is the most commonly used biocide on fishing nets in Japan, accounting for more than 71% of total emissions in the fiscal year 2020 as reported by the Japanese PRTR [2]. This article focuses on the ecological toxicity of the PC that has been widely used on fishing nets over the past two decades.

PC, a dithiocarbamate (DTC) fungicide, was originally developed as a pesticide to control fungal diseases in various field crops [3]. DTCs have long been used for agricultural purposes, and agricultural runoff of these chemicals into surrounding water bodies is inevitable; therefore, their toxicity to freshwater organisms has been evaluated and reported [4,5,6]. However, only a few studies have discussed the toxicity of PC to fish in the marine environment [7,8], and there are no reports on toxicity to algae or crustaceans. Because PC has also been used as an antifouling biocide in coastal waters, its impact on marine organisms is a matter of concern.

**Figure 1:**
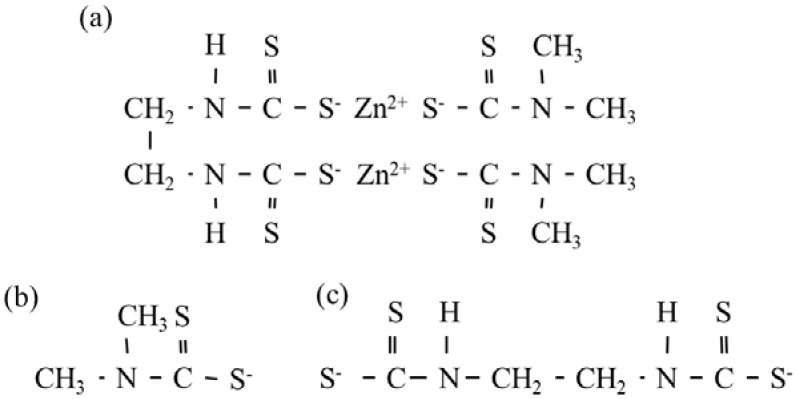
Structural formulas of (**a**) polycarbamate (PC) and its main degradation products, (**b**) dimethyldithiocarbamate (DMDC) and (**c**) ethylenebisdithiocarbamate (EBDC).

Dimethyldithiocarbamate (DMDC) and ethylenebisdithiocarbamate (EBDC) are the main components of PC, considering that PC is formed by two molecules of DMDC and one molecule of EBDC combined with zinc (Figure 1). PC is hydrolyzed into DMDC and EBDC in aquatic environments because the zinc in PC dissolves in water and the cross-links of zinc break. Thus, the toxicity data of DMDC and EBDC are important for performing risk assessments of PC. The toxicity of these compounds to freshwater organisms has been reported [4,5,6], but no studies have investigated the toxicity of these compounds to marine organisms. DMDC has been detected in coastal water (<0.91–43 ng/L) and sediment (<170–1700 ng/kg dry weight) in Japan [9]. Additionally, the concentration of PC in the Chikugo River in Japan (<61–453 ng/L) has been calculated under the assumption that all detected DMDC molecules were derived from PC [10].

The species sensitivity distribution (SSD) approach (developed by the United States Environmental Protection Agency, Washington, D.C., USA [US EPA]) has been employed to evaluate the ecological risks of pesticide pollution by statistically describing the dose-dependent effects of contaminants at the species level [11]. Using this approach, the hazardous concentration that affects 5% of the species (HC_5_) has been identified as an important indicator of aquatic safety; HC_5_ (with a 95% confidence interval) is often recognized as the threshold for aquatic life [12]. To increase the robustness of the indicator, it has been proposed that the HC_5_ should be calculated from the model-averaged SSD obtained from multiple species distributions [13] using an R library [14]. The guidelines for SSD approaches recommend using an uncertainty factor to obtain the predicted no-effect concentration (PNEC) from the HC_5_ values [15,16,17]. The utilization of probabilistic analyses to characterize risks has recently become a popular approach and is now the official method for assessing the ecological risks of chemicals in many countries, including the USA, the European Union, Australia, and New Zealand [18,19,20]. However, the risk posed by PC has not been characterized using probabilistic analysis to date.

The primary aim of this study was to evaluate the acute and chronic toxicities of PC to the marine algae *Dunaliella tertiolecta* Butcher, *Skeletonema marinoi–dohrnii* complex (a complex of *Skeletonema marinoi* Sarno & Zingone and *S. dohrnii* Sarno & Kooistra), and *Tetraselmis tetrathele* (West) Butcher and the crustaceans Japanese blue crabs (*Portunus trituberculatus* Miers) and *Tigriopus japonicus* Mori. In addition, the acute toxicity of DMDC and EBDC was evaluated using the *S. marinoi–dohrnii* complex, which is the most sensitive organism to PC out of all tested organisms. The secondary aim of this study was to assess the ecological risk of PC toxicity. The PNEC was estimated using conventional and probabilistic approaches based on the toxicity values derived in the present study. The predicted environmental concentrations of PC were estimated from the previously reported DMDC concentrations in Hiroshima Bay [9], assuming that all detected DMDC molecules were derived from PC. The ecological risks from the derived PNEC value and the PC concentration were investigated.

## 2. Results

### 2.1. Toxicity

#### 2.1.1. Polycarbamate (PC)

Actual measured concentrations of PC averaged about 12 ± 3% (mean ± standard error) of the targeted nominal concentrations for the algal growth inhibition tests (Table 1). The algal growth inhibition tests using *D. tertiolecta* and the *S. marinoi–dohrnii* complex satisfied the performance criteria described in the Organization for Economic Cooperation and Development (OECD) test guideline [21], i.e., the growth rate of these algae in the control was higher than 0.92/day during the test period. The 50% effective concentration (EC_50_) at 72 h and the no observed effect concentration (NOEC) of PC for *T. tetrathele* were used for reference because the growth rate in the control was lower than 0.92/day during the test period. The 72 h EC_50_ of PC for *D. tertiolecta*, *S. marinoi–dohrnii* complex, and *T. tetrathele* were 3.0, 0.72, and 12 μg/L, respectively. The NOEC of PC for *D. tertiolecta*, *S. marinoi–dohrnii* complex, and *T. tetrathele* were 0.48, 0.45, and 1.2 μg/L, respectively (Table 1).

The actual measured concentrations of PC averaged about 36 ± 2% of the targeted nominal concentrations for the crustacean immobilization tests (Table 2). In the crustacean immobilization tests of PC, the immobilization in the solvent control group was ≤10% (Table 2). The 50% lethal concentration (LC_50_) of PC at 24 h for Japanese blue crab and *T. japonicus* was 16 and 2.0 μg/L, respectively, under dark conditions at 20 °C.

#### 2.1.2. Main Degradation Products of Polycarbamate (PC)

The actual measured concentration of the sodium salt of DMDC (Na-DMDC), which is a main component of PC, averaged about 15 ± 2% of the targeted nominal concentrations for the algal growth inhibition tests. The 72 h EC_50_ and NOEC of Na-DMDC for the *S. marinoi–dohrnii* complex based on the nominal concentration were 5.2 and 3.5 μg/L, respectively, and the values based on the actual concentration were 0.84 and 0.48 μg/L, respectively (Table 3). In the test of the disodium salt of EBDC (2Na-EBDC), another main component of PC, the chemical was not detected at sufficiently high sensitivity in the test solution. Based on the nominal concentration, the 72 h EC_50_ and NOEC of EBDC for the *S. marinoi–dohrnii* complex were 15 and 10 μg/L, respectively (Table 3).

### 2.2. Species Sensitivity Distribution (SSD)

**Figure 2:**
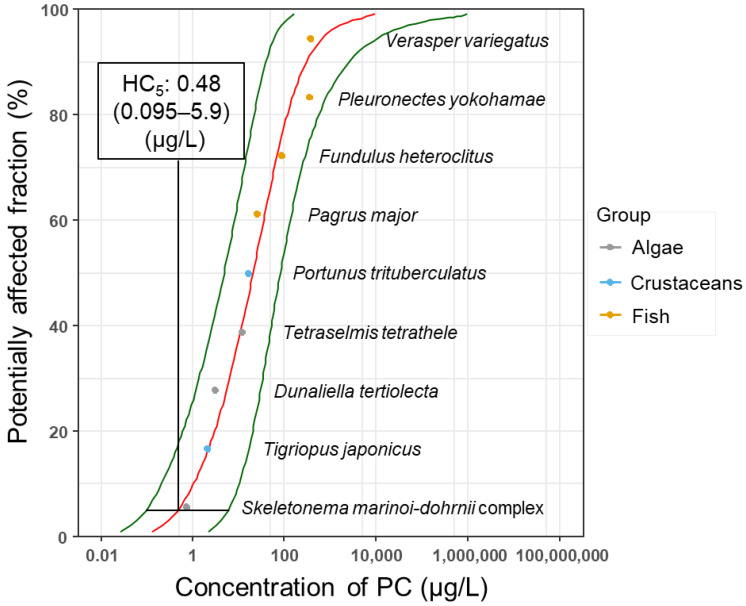
Species sensitivity distributions (SSDs) to polycarbamate (PC) derived from the model using the acute toxicity values (red line). The two green lines indicate the 95% confidence intervals. The positions of the 95% confidence interval of HC_5_ are indicated by horizontal solid bars. The gray, blue, and orange circles represent the toxicity values for algae, crustaceans, and fish, respectively.

The SSD approach for PC was carried out using the acute toxicity data (LC_50_ and EC_50_) obtained in the present study and reported data. When two toxicity values were obtained from one species, the geometric mean of the values was used for SSD analysis (Table 4). Acute toxicity values from nine species (three algal species, two crustacean species, and four teleost fish species) were used for deriving SSDs with the cumulative distribution functions using seven distributions, namely, Weibull, log–logistic, log–normal, gamma, log–normal/log–normal mix, log–logistic/log–logistic mix, and Gumbel, and model-fit statistics were calculated (Table 5). A model-averaged SSD was determined based on the Akaike information criterion corrected for sample size (AICc) values using the R package ssdtools [14] and establishing a model-averaged HC_5_ (Figure 2). The HC_5_ value (0.48 [0.095–5.9] μg/L) was obtained based on the acute toxicity of PC. The results of the Shapiro–Wilk test confirmed the normality of the log-transformed SSD data (*p* = 0.4976 for the acute toxicity values). The SSD approach was not performed using the chronic toxicity values because at least six toxicity values are required for calculations using the R library, but only five chronic toxicity values (NOEC) were obtained from the present study and published data.

## 3. Discussion

### 3.1. Toxicity to Marine Algae and Crustaceans

To the best of our knowledge, this is the first study to evaluate the acute and chronic toxicities of PC to marine algae and crustaceans. The acute and chronic toxicities of PC to marine fish have been reported [7,8], and those to marine organisms are shown in Table 4. Algae, especially the diatom *S. marinoi–dohrnii* complex, are thought to be more sensitive to PC compared to crustaceans and fish, similar to their greater sensitivity to other DTCs [4].

The acute toxicity of ziram, ferbam, and thiram to the freshwater algae *Chlorella pyrenoidosa* I. Shihira & R.W. Krauss was reported to be 1.2, 2.4, and 1.0 μg/L (3.9, 5.8, and 4.2 nM), respectively (96 h EC_50_, [4]). The toxicity of PC to the *S. marinoi–dohrnii* complex (72 h EC_50_: 0.72 μg/L, 1.2 nM) is thought to be similar to those of DTCs. The acute toxicity of ziram, ferbam, and thiram to the freshwater crustacean *Daphnia magna* Straus was reported to be 670, 140, and 210 μg/L (2200, 340, and 870 nM), respectively (48 h EC_50_, [4]). The toxicity of PC to Japanese blue crab (24 h EC_50_: 16 μg/L, 28 nM) and *T. japonicus* (24 h EC_50_: 2.0 μg/L, 3.4 nM) is thought to be higher than those of DTCs, suggesting that marine crustaceans are more sensitive to PC than freshwater crustaceans.

The acute toxicity of Na-DMDC and nabam (2Na-EBDC) to freshwater algae *C. pyrenoidosa* was reported to be 0.8 and 2.4 μg/L (5.4 and 9.4 nM), respectively (96 h EC_50_, [4]). The sensitivity of the *S. marinoi–dohrnii* complex to Na-DMDC (72 h EC_50_: 0.84 μg/L, 5.9 nM, based on actual concentration) and 2Na-EBDC (72 h EC_50_: 15 μg/L, 59 nM, based on nominal concentration) is thought to be similar to and lower than that of *C. pyrenoidosa*, respectively.

Based on the nominal concentration, the toxicity of PC, Na-DMDC, and 2Na-EBDC to the *S. marinoi–dohrnii* complex, which is the most sensitive test organism to PC, was 13, 36, and 59 nM, respectively (Table 1 and Table 2). PC breaks down into two molecules of DMDC and one molecule of EBDC in water, according to the structural formula of PC (Figure 1). The contribution of DMDC and EBDC to the toxicity of PC was estimated based on the concept of concentration addition [22], 13 nM (concentration of PC)/13 nM (EC_50_ of PC) = 13 nM × 2 molecules (concentration of DMDC)/36 nM (EC_50_ of DMDC) + 13 nM (concentration of EBDC)/59 nM (EC_50_ of EBDC) = 0.72 + 0.22 = 0.94. Thus, the toxicities of DMDC and EBDC could almost explain that of PC, and the toxicity of DMDC was thought to contribute the most (72%) to the toxicity of PC based on the results of the algal growth inhibition tests using the *S. marinoi–dohrnii* complex. This study elucidates the toxicity of the degradation products of PC to the marine alga *S. marinoi–dohrnii* complex. The toxicities of these products to other marine organisms, especially crustaceans and fish, should also be elucidated.

### 3.2. Derivation of Predicted No-Effect Concentration (PNEC)

To the best of our knowledge, this is the first study to estimate the PNECs of PC using probabilistic techniques. Toxicity values from a sufficiently large and ecologically diverse sample of species are needed to estimate the PNEC reliably. In the present study, SSD analyses based on acute toxicity values were carried out using ecotoxicity data obtained from nine species. In addition, the acute toxicity values include the values from species belonging to three trophic levels. The number of toxicity values required to determine SSD varies between guidelines from each nation (e.g., greater than or equal to four through eight) [16,17,18]. Five or more toxicity values are essential for SSD analysis if the ecotoxicity data assumed to follow a log-normal distribution [23] are both cost-effective and high-performance enough to be included in the analysis [24]. At least six toxicity values are required for calculations using the R library ssdtools [14]. Therefore, the HC_5_ obtained in the present SSD analysis is considered appropriate for assessing the ecological risk of PC. The derivation of a value corresponding to PNEC from HC_5_ varies between the guidelines. HC_5_ itself is seldom used as a PNEC. Although an uncertainty factor of 1–5 is applied to HC_5_ [15,16,17] in many cases, a default factor of 10 is applied to HC_5_ derived from acute toxicity values [16]. Overall, the factor is determined by experts by considering the number of toxicity values and composition of species used in the SSD analysis [15,16]. In the present study, an HC_5_ of 0.48 μg/L was obtained from the acute toxicity values. The uncertainty factor of 10 was applied to the HC_5_. A PNEC of 0.048 μg/L was estimated through the probabilistic approach.

The chronic toxicity of PC for marine organisms from two trophic levels was determined, and the lowest toxicity was 0.45 μg/L for the marine alga *S. marinoi–dohrnii* complex. Conventionally, an uncertainty factor of 50 or 100 is usually applied when chronic toxicity values are obtained from two trophic levels, according to some guidelines, such as the Chemical Substances Control Law in Japan [25] and OECD guidelines [26]. In the present study, 100 was used as the uncertainty factor because this is the most conservative value in the range of recommended values. Thus, the PNEC was calculated to be 0.0045 μg/L. In addition, the toxicity of DMDC to a marine alga was 0.48 μg/L when the same uncertainty factor (100) was adopted, and the reference PNEC was calculated to be 0.0048 μg/L.

### 3.3. Ecological Risk Assessment of Polycarbamate (PC)

In this study, the PNEC of PC for marine organisms was derived using both conventional and probabilistic approaches. Assuming that the detected analyte was derived from PC, our recent investigations estimated the maximum PC concentration in seawater to be 0.11 μg/L in Hiroshima Bay [9], which is higher than the PNECs derived using both the conventional and probabilistic approaches. The maximum DMDC concentration in seawater was reportedly 0.043 μg/L in Hiroshima Bay [9], which is higher than the PNECs obtained using the conventional approach. The ecological risk of PC and DMDC contamination in the Seto Inland Sea is thus currently considered to be high. DTCs including PC are thought to be more stable in seawater than in freshwater because the half-lives of DTCs in solutions increase with increased pH [4]. PCs have been mainly used to coat fishing nets in marine environments [1]. Therefore, the ecological risk of DTCs in the marine environment may be higher than in the freshwater environment.

### 3.4. Scope for Future Research

This study demonstrated that PC is highly toxic to some algae and crustaceans but did not examine the chronic effects of PC on crustaceans or the effects of PC on algae other than microalgae (e.g., macroalgae). The effects of PC on these organisms need to be studied in the future. As DTCs in the environment are broken down into DMDC and EBDC, it is difficult to analyze undegraded DTCs separately, and it is not possible to assess the ecological risk of each DTC without preconditions. Therefore, the toxicity of the degradation products of DTCs such as DMDC and EBDC to marine organisms must be clarified and the ecological risk assessed in more detail.

## 4. Materials and Methods

### 4.1. Organisms

The test organisms used in this study included algae (*D. tertiolecta*, *T. tetrathele*, and *S. marinoi-dohrnii* complex [NIES-324]) and crustaceans (*T. japonicus* and Japanese blue crab [*P. trituberculatus*]). Nauplii of *T. japonicus* (<24 h after hatching) and hatched zoeae of Japanese blue crabs were used in the crustacean immobilization tests.

*Dunaliella tertiolecta* and *T. tetrathele* were provided by the Shiogama Branch, Japan Fisheries Research and Education Agency (Shiogama, Japan), and the *S. marinoi–dohrnii* complex was purchased from the National Institute for Environmental Studies (NIES-324; Tsukuba, Japan). Each algal species was maintained according to the procedure described by Onduka et al. [27] in f/2 medium [28] under static-renewal conditions on a 14:10 h light:dark cycle (light intensity: 39 ± 8 μmol/m^2^/s in the wavelength range 360–670 nm) in a temperature-controlled (20 ± 1 °C) growth chamber (MLR-350; Sanyo, Osaka, Japan). The f/2 growth medium was prepared from natural seawater filtered through sand, activated carbon, and a glass fiber filter (GF/C, Whatman, Maidstone, UK; hereafter referred to as “filtered seawater”).

Adult *T. japonicus* were obtained from the Marine Ecology Research Institute (Tokyo, Japan) and cultured, and *T. japonicus* nauplii (age < 24 h) from this culture were collected in a 1 L glass beaker containing filtered seawater as described by Onduka et al. [29]. The culture conditions were the same as those used for culturing algae, except that cultured *T. tetrathele* was added as food. Japanese blue crabs carrying eggs were obtained from the Hamasui Fisheries Company (Hiroshima, Japan). The eggs were placed in glass Petri dishes (90 mm in diameter) containing filtered seawater and incubated at 20 °C overnight with gentle shaking. The hatched zoeae were then used for the toxicity tests.

### 4.2. Chemicals

PC (molecular weight, 581.56; purity, >95.1%), and DMDC-methyl (purity, 100%) were obtained from Wako Pure Chemical Industries Ltd. (Osaka, Japan). Na-DMDC (molecular weight, 143.21; purity, >98.0%) and 2Na-EBDC (molecular weight, 256.34; purity, 99.5%) were obtained from Tokyo Chemical Industry Co., Ltd. (Tokyo, Japan) and Sigma–Aldrich (St. Louis, MO, USA), respectively. Stock solutions of PC, Na-DMDC, and 2Na-EBDC were prepared by diluting the compounds with dimethyl sulfoxide (DMSO; plant cell culture-tested DMSO, Sigma–Aldrich). Acetic acid was purchased from Nacalai Tesque Inc. (special grade, Kyoto, Japan). Other chemicals were obtained from Wako Pure Chemical Industries unless otherwise noted.

### 4.3. Toxicity Tests

Toxicity tests were performed by following the test guidelines of the OECD [21,30] that were modified for marine organisms. Algal growth inhibition tests and crustacean immobilization tests were carried out as described by Onduka et al. [27,31].

#### 4.3.1. Algae

To perform the algal growth inhibition tests, test solutions were prepared by diluting the stock solution (1000 mg/L) and DMSO (solvent control) 3000 times with f/2 medium. The algal growth inhibition tests were carried out in triplicate with a geometric series of nominal PC, Na-DMDC, and 2Na-EBDC concentrations (Table 1 and Table 3). The assays were performed using f/2 medium under the same photoperiod and temperature conditions used for the algal cultures and a pH of 8.6 ± 0.6, as described by Onduka et al. [27]. The light source consisted of three ultraviolet-screened fluorescent tubes that yielded a photosynthetic photon flux (360–670 nm) of 41 ± 7 μmol/m^2^/s. During the algal growth inhibition test, the specific growth rate (μ; d^−1^) was calculated as μ = 1/3 × ln (N_72h_/N_0h_), where N_0h_ is the initial cell concentration, and N_72h_ is the cell concentration after 72 h. The cell concentration was calculated from the relationship between the in vivo fluorescence and the concentration of the alga. The percentage inhibition of growth rate (I) for each treatment was calculated as I = (μ_c_ − μ_t_)/μ_c_ × 100, where μ_c_ and μ_t_ are the average specific growth rates in the control and exposure groups, respectively. A percentage inhibition of more than 100% means that the cell concentration after 72 h was lower than the initial cell concentration.

#### 4.3.2. Crustaceans

To perform the crustacean immobilization tests, test solutions were prepared by diluting the stock solution (1000 mg/L) and DMSO (solvent control) 2000 times with filtered seawater. The crustacean immobilization tests were performed in a glass weighing bottle (φ25 × 25 mm, 6 mL). Five nauplii or zoeae were placed in a well containing 2 mL of filtered seawater. Four wells were prepared for each concentration of the test substances. The tests were carried out using a geometric series of nominal PC concentrations (Table 2). Animals were not fed during the 24 h test period. The tests were conducted in the dark at 20 °C. Immobilization, which is defined as the inability to swim within 15 s after gentle agitation, was checked at the end of the test. The pH range of the test solutions was 8.0 ± 0.0.

### 4.4. Chemical Analysis

PC and Na-DMDC in the test solutions were analyzed based on the decomposition of PC and Na-DMDC to DMDC followed by methyl derivatization to DMDC-methyl [9]. Water samples were collected and analyzed for the test chemical at 0 and 72 h for the algal growth inhibition test and at 0 and 24 h for the crustacean immobilization tests. The test solutions at the end of the algal growth inhibition test and crustacean immobilization tests were pooled for each exposure group. The test solutions were filtered through a GF/C filter (GE Healthcare, Little Chalfont, UK), and 50 mL (for the algal growth inhibition tests) or 5 mL (for the crustacean immobilization tests) of the solution underwent chemical analysis. The derivatization, extraction, and clean-up methods for PC and Na-DMDC followed the methods described by Hano et al. [9]. Working standard solutions of DMDC-methyl were prepared by diluting the stock solution (100 µg/L in hexane) with hexane. Anthracene-d_10_ was used as an internal standard. Elution was performed by adding the internal standard (anthracene-d_10_; final concentration, 50 µg/L) to each working solution, concentrating them at 40 °C under gentle nitrogen gas to prepare final solutions in 0.5 mL of hexane, and subjecting these solutions to gas chromatography with mass spectrometric detection (GC-MSD; 6890N, 5975 inert; Agilent Technologies, Tokyo, Japan).

For gas chromatographic separation, a 30 m × 0.25 mm (inner diameter) capillary column (HB-5MS; Agilent Technologies) was used. The column was maintained at 60 °C for 2 min, followed by programmed heating to 220 °C at a rate of 20 °C/min. The helium gas flow rate during this procedure was 1.0 mL/min. The mass spectrometer was operated under selected-ion monitoring mode using molecular ions of the target test chemicals (electron impact at 70 eV, ion source temperature, 230 °C). The interface temperature was 275 °C. The ions (m/z) monitored in this mode were DMDC-methyl (88, 135) and anthracene-d_10_ (188).

PC concentrations were recalculated from the DMDC-methyl concentrations. For PC analysis, the concentration of PC (p) was calculated as p = d × 0.89/0.41, where d is the concentration of DMDC-methyl [9]. The detection limit (DL) was determined as DL = 10 × signal-to-noise ratio. The DL of DMDC-methyl and PC for 50 mL of the algal test solution were 0.02 and 0.04 µg/L, respectively. The detection limit of the PC for 5 mL of the crustacean test solution was 0.2 µg/L. The geometric mean of the toxicant concentrations was used to estimate the EC_50_, lowest observed effect concentration (LOEC), and NOEC.

### 4.5. Data Analysis

The 24 h and 72 h EC_50_ were estimated, and multiple comparison tests were carried out using R software [32] (version 4.2.1), primarily with the drc and multcomp (Dunnett’s test) packages [33,34]. Differences between groups were considered significant at *p* < 0.05. The normality of the log-transformed SSD data was confirmed using R software with the dplyr (Shapiro–Wilk test) package [35].

To develop the SSD curves and estimate the HC_5_ values for PC, the maximum likelihood estimation (MLE): model-averaging approach was used [13,36]. This technique is robust because more than one curve can fit the data equally well in some instances, and the choice of the curve impacts the calculation of the HC_5_. By using the model-averaging approach, bias caused by the selection of one curve over another can be avoided. Moreover, the resulting weighted distribution is relatively stable and not greatly influenced by small changes in the dataset [13,36].

The methods and recommendations outlined by Schwarz and Tillmanns [13] and R [32] (version 4.2.1) with R Studio [37] (version 2022.12.0) and the R package ssdtools [14] (version 1.0.2) were used to implement the following cumulative distribution functions based on their ability and robustness of the fitted SSD data: Weibull, log–logistic (llogis), log–normal (lnorm), gamma, log–logistic/log–logistic mix (lnorm_lnorm) log–normal/log–normal mix (lnorm_lnorm), and Gumbel (lgumbel). The quality of the fitted distributions using MLE as the regression method was then assessed for each scenario using the AICc, which is a measure of the relative quality of fit to the dataset, for each distribution [13,38,39]. A model-averaged SSD was determined based on AICc values of multiple distributions using the R package ssdtools [14], and a model-averaged HC_5_ was estimated using the model-averaged SSD [13,36]. Parametric bootstrap resampling (using 10,000 iterations) was then performed to calculate confidence intervals across the entire SSD curve, including the HC_5_ [13,36].

## 5. Conclusions

In this study, the acute and chronic toxicities of PC to marine organisms such as algae and crustaceans were evaluated under laboratory conditions. It was determined that algae, especially the diatom *S. marinoi–dohrnii* complex, are more sensitive to PC than crustaceans and fish are. In addition, we evaluated the acute toxicity of DMDC and EBDC, which are the main degradation products of PC, to the *S. marinoi–dohrnii* complex, the most sensitive to PC out of all tested organisms, and demonstrated that the toxicity of DMDC is similar to that of PC and plays a large role in the toxicity of PC. The PNEC of PC for marine organisms was derived through probabilistic and conventional approaches. With regards to the ecological risk of PC, the highest concentration of PC in Hiroshima Bay was 0.11 μg/L, which is higher than the PNECs derived through both the conventional and probabilistic approaches. Therefore, the ecological risk of PC contamination in Hiroshima Bay is currently considered to be high. Hence, stakeholders should formulate strategies for minimizing the risks posed by DTCs including PC, such as, for example, restricted use of DTCs in the bay and the basin of the river running into the bay.

## Figures and Tables

**Table 1:** Toxicities of polycarbamate (PC) to *Dunaliella tertiolecta*, *Skeletonema marinoi–dohrnii* complex, and *Tetraselmis tetrathele*.

Organisms	Concentration (mg/L)				Cell Density (×10^5^ cells/mL)		Growth Rate (/d)	Growth Inhibition	72 h EC_50_^a^	72 h LOEC	72 h NOEC
	Nominal	Actual									
		0 h	72 h	Geometric Mean	0 h	72 h	0–72 h	(%)	(μg/L)	(μg/L)	(μg/L)
*Dunaliella tertiolecta*	Control	<0.04	<0.04	<0.04	1.0 ± 0.0	16.5 ± 0.3	0.93 ± 0.01	1 ± 1			
	0 (SC)	<0.04	<0.04	<0.04	1.0 ± 0.0	17.0 ± 0.2	0.94 ± 0.01	0 ± 1			
	2.5	0.34	0.05	0.13	1.0 ± 0.0	15.9 ± 0.2	0.92 ± 0.00	3 ± 0	3.0	0.94	0.48
	5	1.00	0.23	0.48	1.0 ± 0.0	14.3 ± 0.3	0.88 ± 0.01	7 ± 1	(2.5–3.5)	[1.6]	[0.83]
	10	2.04	0.43	0.94	1.0 ± 0.0	10.0 ± 0.7	0.76 ± 0.02	19 ± 2 *	[5.2 (4.3–6.0)]		
	20	4.63	1.33	2.48	1.0 ± 0.0	6.7 ± 0.6	0.62 ± 0.03	35 ± 3 *			
	40	11.05	3.19	5.94	1.1 ± 0.0	1.9 ± 0.2	0.17 ± 0.04	82 ± 4 *			
*Skeletonema marinoi–dohrnii* complex	Control	<0.04	<0.04	<0.04	1.1 ± 0.0	85.8 ± 5.0	1.45 ± 0.02	5 ± 1			
	0 (SC)	0.07	<0.04	<0.04	1.1 ± 0.0	109.6 ± 2.8	1.53 ± 0.01	0 ± 1			
	3.5	0.81	0.11	0.29	1.1 ± 0.0	106.8 ± 1.2	1.53 ± 0.00	0 ± 0	0.72	0.65	0.45
	5	1.40	0.14	0.45	1.1 ± 0.0	94.3 ± 4.6	1.50 ± 0.02	2 ± 1	(0.63–0.82)	[1.1]	[0.77]
	7	1.77	0.24	0.65	1.1 ± 0.0	29.9 ± 9.3	1.06 ± 0.10	30 ± 7 *	[1.2 (1.1–1.4)]		
	10	3.11	1.77	2.34	1.2 ± 0.0	1.0 ± 0.3	−0.11 ± 0.14	108 ± 9 *			
	14	5.24	4.15	4.66	1.3 ± 0.0	0.2 ± 0.0	−0.68 ± 0.01	145 ± 1 *			
*Tetraselmis tetrathele*	Control	<0.04	<0.04	<0.04	0.85 ± 0.07	13.1 ± 1.0	0.89 ± 0.02	2 ± 2			
	0 (SC)	<0.04	<0.04	<0.04	0.85 ± 0.01	13.2 ± 0.2	0.91 ± 0.01	0 ± 1			
	5	2.36	0.57	1.16	0.86 ± 0.01	12.6 ± 0.3	0.89 ± 0.01	3 ± 1	12 ^b^	2.4	1.2
	10	4.55	1.26	2.39	0.87 ± 0.01	9.2 ± 0.6	0.77 ± 0.02	15 ± 2 *	(10–14)	[4.1]	[2.1]
	20	10.23	4.59	6.85	0.98 ± 0.02	5.5 ± 0.1	0.58 ± 0.00	37 ± 0 *	[21 (17–24)]		
	40	20.26	9.26	13.70	1.25 ± 0.06	4.2 ± 0.2	0.41 ± 0.01	55 ± 1 *			
	80	39.10	22.48	29.64	1.25 ± 0.12	3.0 ± 0.3	0.27 ± 0.03	70 ± 4 *			

Data are expressed as the mean ± standard error. * Significantly different (*p* < 0.05) from the value for the solvent control. ^a^: The 95% confidence intervals are given in parentheses. ^b^: This value was considered the reference value because growth rate in the control was lower than 0.92/day. SC: solvent control; EC_50_: 50% effective concentration; LOEC: lowest observed effect concentration; NOEC, no observed effect concentration. Toxicity values in nanomolar concentrations are given in square brackets.

**Table 2:** Acute toxicities of polycarbamate (PC) to zoeae (<24 h post hatching) of Japanese blue crab (*Portunus trituberculatus*) and nauplii (<24 h post hatching) of *Tigriopus japonicus*.

Organisms	Concentration (mg/L)				Number of Organisms		24 h EC_50_ ^a^
	Nominal	Actual					
		0 h	24 h	Geometric Mean	Exposed	Immobilization	(μg/L)
Japanese blue crab	Control	<0.2	<0.2	<0.2	20	2	
	0 (SC)	<0.2	<0.2	<0.2	20	2	
	10	7.2	2.79	4.5	20	2	
	20	13	4.3	7.4	20	7	16
	40	25	7.0	13	20	12	(6.2–25)
	80	48	22	32	20	12	[28 (11–43)]
	160	80	27	46	20	14	
	320	166	31	72	20	16	
*Tigriopus japonicus*	Control	<0.2	<0.2	<0.2	20	0	
	0 (SC)	0.6	0.2	0.3	20	1	
	2.5	1.7	0.5	0.9	18	4	2.0
	5	3.1	1.6	2.2	21	10	(1.4–2.6)
	10	6.5	2.5	4.0	20	17	[3.4 (2.4–4.5)]
	20	12.0	3.6	6.5	20	20	
	40	23.2	7.7	13.3	20	20	

^a^: The 95% confidence intervals are given in parentheses. SC: solvent control EC_50_: 50% effective concentration. Toxicity values in nanomolar concentrations are given in square brackets.

**Table 3:** Toxicities of the main degradation products of polycarbamate (PC) to the *Skeletonema marinoi–dohrnii* complex.

Tested Chemical	Concentration (mg/L)				Cell Density (×10^5^ cells/mL)		Growth Rate (/d)	Growth Inhibition	72 h EC_50_^a^	72 h LOEC	72 h NOEC
	Nominal	Actual									
		0 h	72 h	Geometric Mean	0 h	72 h	0–72 h	(%)	(mg/L)	(mg/L)	(mg/L)
Na-DMDC	Control	<0.02	<0.02	<0.02	1.1 ± 0.0	85.8 ± 5.0	1.45 ± 0.02	0 ± 1			
	0 (SC)	0.10	<0.02	<0.02	1.2 ± 0.0	95.0 ± 2.9	1.45 ± 0.01	0 ± 1	5.2 (4.8–5.6) ^c^	5.0 ^c^	3.5 ^c^
	2.5	0.41	0.07	0.17	1.2 ± 0.0	89.7 ± 2.4	1.45 ± 0.01	0 ± 1	[36 (34–39) ^c^]	[35 ^c^]	[24 ^c^]
	3.5	0.81	0.28	0.48	1.2 ± 0.0	75.6 ± 14.8	1.38 ± 0.08	5 ± 5			
	5	1.64	0.38	0.79	1.1 ± 0.0	21.6 ± 8.2	0.93 ± 0.12	36 ± 8 *	0.84 (0.77–0.92) ^d^	0.79 ^d^	0.48 ^d^
	7	1.96	0.92	1.35	1.2 ± 0.0	0.9 ± 0.1	−0.07 ± 0.03	105 ± 2 *	[5.9 (5.4–6.4) ^d^]	[5.5 ^d^]	[3.4 ^d^]
	10	2.30	1.90	2.09	1.3 ± 0.0	0.3 ± 0.0	−0.46 ± 0.02	131 ± 1 *			
2Na-EBDC ^b^	Control				1.1 ± 0.0	85.8 ± 5.0	1.45 ± 0.02	0 ± 1			
	0 (SC)				1.0 ± 0.0	86.1 ± 3.5	1.46 ± 0.02	0 ± 1			
	7				1.0 ± 0.0	71.1 ± 5.2	1.40 ± 0.03	4 ± 1	15 (12–18) ^c^	14 ^c^	10 ^c^
	10				1.0 ± 0.0	61.3 ± 6.5	1.36 ± 0.04	7 ± 3	[59 (47–70) ^c^]	[55 ^c^]	[39 ^c^]
	14				1.0 ± 0.0	42.3 ± 6.3	1.23 ± 0.05	16 ± 3 *			
	20				1.0 ± 0.0	0.4 ± 0.1	−0.33 ± 0.07	122 ± 5 *			
	28				1.0 ± 0.0	0.1 ± 0.0	−0.67 ± 0.02	146 ± 1 *			

Data are expressed as the mean ± standard error. * Significantly different (*p* < 0.05) from the value for the solvent control. Na-DMDC: sodium diethyldithiocarbamate; 2Na-EBDC: disodium ethylenebisdithiocarbamate; SC: solvent control; EC_50_: 50% effective concentration; LC_50_: 50% lethal concentration; LOEC: lowest observed effect concentration; NOEC: no observed effect concentration. Toxicity values in nanomolar concentrations are given in square brackets. ^a^: The 95% confidence intervals are given in parentheses. ^b^: The chemical was not detected at sufficiently high sensitivity during the test. ^c^: The values are estimated based on the nominal concentration. ^d^: The values are estimated based on the actual concentration.

**Table 4:** Toxicity of polycarbamate (PC) to various marine organisms, including algal species, crustaceans, and teleosts, calculated by performing species sensitivity distribution analysis.

Taxon	Species	Endpoint	Toxicity	Geometric Mean
Algae	*Dunaliella tertiolecta*	72 h EC_50_	3.0 (2.2–3.7) μg/L	
		72 h NOEC	0.48 μg/L	
	*Skeletonema marinoi–dohrnii* complex	72 h EC_50_	0.72 (0.63–0.82) μg/L	
		72 h NOEC	0.45 μg/L	
	*Tetraselmis tetrathele*	72 h EC_50_	12 (10–14) μg/L	
		72 h NOEC	1.2 μg/L	
Invertebrates				
Crustaceans	Japanese blue crab	24 h EC_50_	16 (6.2–25) μg/L	
	(*Portunus trituberculatus*)			
	*Tigriopus japonicus*	24 h EC_50_	2.0 (1.4–4.5) μg/L	
Vertebrates				
Teleost fish	Marbled flounder	96 h LC_50_	301 (190–413) μg/L ^a^	331 mg/L
	(*Pseudopleuronectes yokohamae*)		363 (153–575) μg/L ^a^	
	Mummichog	96 h LC_50_	12 (10–14) μg/L ^b,c^	88 μg/L
	(*Fundulus heteroclitus*)		13 (8.2–22) μg/L ^b,c^	
			540 (220–870) μg/L ^b,d^	
			710 (670–760) μg/L ^b,d^	
		55 d LOEC	3.9 μg/L ^b^	
		55 d NOEC	2.1 μg/L ^b^	
		MATC	2.9 μg/L ^b^	
	Red sea bream	96 h LC_50_	29.2 (11.2–47.3) μg/L ^a^	25.5 μg/L
	(*Pagrus major*)		22.4 (9.9–35.0) μg/L ^a^	
	Spotted halibut	96 h LC_50_	239 (121–356) μg/L ^a^	363 μg/L
	(*Verasper variegatus*)		553 (552–554) μg/L ^a^	
		10 d EC_50_	8.1 (7.5–8.8) μg/L ^b^	
		10 d LOEC	8.5 μg/L ^b^	
		10 d NOEC	6.7 μg/L ^b^	

The 95% confidence intervals are given in parentheses. EC_50_: 50% effective concentration; LC_50_: 50% lethal concentration; MATC: maximum acceptable toxicant concentration; LOEC: lowest observed effect concentration; NOEC: no observed effect concentration. ^a^: Hano et al., 2017 [7]. ^b^: Mochida et al., 2018 [8]. ^c^: larval fish. ^d^: juvenile fish.

**Table 5:** Model-fit statistics used to estimate the fifth percentile of the hazard concentrations derived from the species sensitivity distribution based on the acute toxicity of PC shown in Figure 2.

Distribution	Anderson Darling	Kolmogorov–Smirnov	Cramer–von Mises	Akaike’s Information Criterion (AIC)	Corrected AIC (AICc)	Bayesian Information Criterion	Delta AIC	AICc Weight
gamma	0.456	0.217	0.0740	96.2	98.2	96.6	1.23	0.145
lgumbel	0.237	0.153	0.0337	95.3	97.3	95.7	0.406	0.218
llogis	0.234	0.138	0.0301	95.7	97.7	96.1	0.787	0.180
llogis_llogis	0.159	0.138	0.0243	91.8	112	92.8	14.9	0.00
lnorm	0.245	0.141	0.0315	94.9	96.9	95.3	0.00	0.267
lnorm_lnorm	0.160	0.139	0.0237	91.0	111	92.0	14.0	0.00
weibull	0.334	0.168	0.0470	95.6	97.6	96.0	0.692	0.189

gamma: gamma distribution; lgumbel: Gumbel distribution; llogis: log–logistic distribution; llogis_llogis: log–logistic/log–logistic mixture distribution; lnorm: log–normal distribution; lnorm_lnorm: log–normal/log–normal mixture distribution; weibull: Weibull distribution.

## Data Availability

The data generated in this study are available within the article.
